# Pupil size and pupil change correlate with distinct cortical neurochemical systems across the adult lifespan

**DOI:** 10.64898/2026.08.24.746786

**Published:** 2026-10-07

**Authors:** Elizabeth Riley, Eve De Rosa, Adam Anderson

## Abstract

Pupillometry is often used as a proxy for locus coeruleus (LC) activity but whether this relationship changes across the lifespan is unknown. We collected simultaneous pupillometry and multi-echo fMRI in 89 adults aged 19-78 during a visual oddball task. Oddball-evoked pupillary responses were present in all age groups and largest in middle age. Oddball-evoked LC BOLD responses were detected only in younger adults and did not predict individual pupillary responses. Pupil size and its first derivative (pupil change) correlated with whole-brain BOLD on distinct timescales and in distinct spatial patterns. Against 19 PET maps, pupil derivative coupling correlated uniquely with norepinephrine transporter (NET) density, and pupil size-unique coupling with μ-opioid, cannabinoid CB1, and glutamatergic mGluR5 density. Only the pupil derivative-NET relationship weakened with age, though it remained significant in every group. Pupil size and pupil change therefore carry different information, and only pupil change correlates with activity in NET-rich regions across the lifespan.

## Full Text Availability

The license terms selected by the author(s) for this preprint version do not permit archiving in PMC. The full text is available from the preprint server.

